# Biomechanical rupture risk assessment of abdominal aortic aneurysms using clinical data: A patient-specific, probabilistic framework and comparative case-control study

**DOI:** 10.1371/journal.pone.0242097

**Published:** 2020-11-19

**Authors:** Lukas Bruder, Jaroslav Pelisek, Hans-Henning Eckstein, Michael W. Gee

**Affiliations:** 1 Mechanics & High Performance Computing Group, Technical University of Munich, Garching, Germany; 2 Department of Vascular Surgery, University Hospital Zurich, Zurich, Switzerland; 3 Clinic for Vascular and Endovascular Surgery, Technical University of Munich, Munich, Germany; Imperial College London, UNITED KINGDOM

## Abstract

We present a data-informed, highly personalized, probabilistic approach for the quantification of abdominal aortic aneurysm (AAA) rupture risk. Our novel framework builds upon a comprehensive database of tensile test results that were carried out on 305 AAA tissue samples from 139 patients, as well as corresponding non-invasively and clinically accessible patient-specific data. Based on this, a multivariate regression model is created to obtain a probabilistic description of personalized vessel wall properties associated with a prospective AAA patient. We formulate a probabilistic rupture risk index that consistently incorporates the available statistical information and generalizes existing approaches. For the efficient evaluation of this index, a flexible Kriging-based surrogate model with an active training process is proposed. In a case-control study, the methodology is applied on a total of 36 retrospective, diameter matched asymptomatic (group 1, *n* = 18) and known symptomatic/ruptured (group 2, *n* = 18) cohort of AAA patients. Finally, we show its efficacy to discriminate between the two groups and demonstrate competitive performance in comparison to existing deterministic and probabilistic biomechanical indices.

## 1 Introduction

An abdominal aortic aneurysm (AAA) is a slowly progressing vascular disease, causing an enlargement of the infrarenal aorta and is considered pathological if the aortic diameter exceeds 30 mm [1]. AAA prevalence has been reported within a range of 1.2% to 3.3% in men older than 60 years based on several studies in western societies [2]. In most cases, AAAs develop asymptomatically over several years, but they can rapidly turn into a serious clinical emergency in case of rupture. More than 50% of patients with a ruptured AAA die before reaching the hospital [1] and perioperative mortality rates range from 40% to 60% [3].

To prevent such a disastrous scenario, the clinical guidelines from the US-based Society for Vascular Surgery recommend elective repair for AAA patients with an aortic diameter greater or equal to 55 mm, regular screening intervals for patients with smaller-sized AAAs and one-time screenings for AAAs in men and women above a certain age and based on established risk factors [1]. This maximum diameter recommendation is based on a risk assessment, where the risk of rupture is weighed against the mortality risk of an elective repair. While the latter risks are relatively well-known, aneurysm rupture is a complex biomechanical failure event. With the increasing use of endovascular repair (EVAR) over open surgical repair (OSR) [4], however, which can be attributed to the significant short term mortality benefit of EVAR (1.4% compared to 4.2%) [1], interventional risks have become a less important factor in the risk assessment process.

Nonetheless, a biomechanical rupture risk assessment can provide an additional important piece of information. It enables the possibility to provide patient-specific screening guidelines, avoid unnecessary interventions [5] and support the clinical decision process for cases that are not covered by the clinical guidelines. The Society for Vascular Surgery’s 55 mm recommendation, e.g., only holds for patients “at low or acceptable surgical risk with a fusiform AAA” [1]. Furthermore, there are no clear or only weak recommendations for women with AAAs of size 50-54 mm, aneurysms with non-fusiform geometries, smaller AAAs [6], or patients at higher surgical risk. In addition to that, not all AAAs are suitable for EVAR, with higher complication rates for AAA cases that are not covered by the instructions for use [7]. Lastly, recent meta-studies (e.g. [8, 9]) on the long term outcomes of EVAR versus OSR could not detect any differences with regards to the all-cause mortality or even concluded in favor of OSR.

In this paper, we present a highly personalized, probabilistic framework for the biomechanical quantification of AAA rupture risk. The framework builds upon a comprehensive database, consisting of tensile experiments that were carried out on 305 AAA tissue samples from 139 patients and corresponding non-invasively and clinically accessible patient data. The approach consistently incorporates the available statistical information in terms of probability distributions in order to account for patient-specific uncertainties about relevant vessel wall properties. We emphasize the importance of accounting for these uncertainties and demonstrate that this leads to a more accurate individualized rupture risk assessment as compared to deterministic approaches.

Our work builds upon previous efforts by our group and collaborators regarding the biomechanical modeling and characterization of AAA in-vivo behavior [10–16], as well as several previous studies indicating that biomechanical indices are more accurate predictors for AAA rupture risk than the clinically established maximum diameter criterion [17–24]. In contrast to the approaches in [17–20, 22, 24], however, we advocate a probabilistic treatment to account for uncertain vessel wall properties. Our work thus goes along the lines of [21], but with the key difference that it includes the stiffness parameters of the AAA vessel wall as statistical quantities, uses patient-specific vessel wall properties and accounts for statistical correlations among these properties.

The paper is organized as follows. Motivated by a failure-based criterion, our rupture risk index is formulated in Section 2.1 incorporating patient-specific statistical information. Section 2.2 defines the biomechanical AAA model and specifies the probabilistic regression model to obtain personalized vessel wall properties. In Section 2.3, a method for the efficient evaluation of the rupture risk index is proposed and in Section 3, the framework is applied on a total of 36 retrospective, diameter matched asymptomatic (group 1, *n* = 18) and known symptomatic/ruptured (group 2, *n* = 18) cohort of AAA patients.

## 2 Materials and methods

### 2.1 Failure-based probabilistic quantification of rupture risk

#### 2.1.1 Rupture as an event of material failure

From a mechanical point of view, rupture represents an event of local material failure at a point **x** in the aneurysm wall, which motivates its definition via a failure function *φ*(**x**) and the failure criterion

$$\begin{array}{c} \varphi (x)>0,\;\text{at}\;\text{any}\;x. \end{array}$$ (1)

We limit ourselves to stress-based failure and define rupture as an event where the local wall stress measure *σ*(**x**) exceeds the local wall strength *σ*_*γ*_(**x**). This results in the failure function *φ*(**x**) = *σ*(**x**) − *σ*_*γ*_(**x**), or the criterion

$$\begin{array}{c} \sigma \left(x\right)>\sigma_{\gamma }\left(x\right),\;\text{at}\;\text{any}\;x. \end{array}$$ (2)

Using the equivalent von Mises stress σ_*vm*_(**x**) as the local stress measure *σ*(**x**) and an assumed spatially constant wall strength *σ*_*γ*_, this criterion can be evaluated as

$$\begin{array}{c} \sigma_{\text{vm}}^{\text{max}}>\sigma_{\gamma }, \end{array}$$ (3)

where $\sigma_{vm}^{max}$ is the maximum von Mises stress $\sigma_{vm}^{max}={\text{max}}_{x}\sigma_{vm}(x)$.

It is important to note that the above definition does not incorporate any aspect about failure over time. In order to be able to include time in the analysis, i.e. to make a statement about the risk of rupture in the next year, one would require knowledge about the future progression of the AAA for this patient, such as a model for the aneurysm growth and change in vessel wall properties. Since there is hardly any knowledge about these aspects, we limit the further discussion to a rupture risk assessment at the point of time of the acquired data. While there are sudden events like calcification-induced formation of saccular aneurysms, we assume that in most cases an AAA is a slowly progressing disease and thus our approach has, at least for the near future, sufficient predictive capability.

#### 2.1.2 Existing criteria and rupture risk indices

Rupture risk estimation for AAAs has been an ongoing research topic over several decades, with many attempts to establish decision criteria for clinical practice. The maximum diameter criterion [1] still represents the most widely used criterion for decision making today. It is often justified by Laplace’s law, which states that the vessel wall stress is proportional to its diameter in spherical geometries. Based on this and with data obtained from several clinical studies, a very simple criterion,

$$\begin{array}{c} d>d_{\text{max}}, \end{array}$$ (4)

has been formulated, relating the patient’s AAA diameter *d* to a critical maximum diameter *d*_max_. While established in clinical practice and easy to apply using CT or ultrasound imaging, this criterion has often been criticized [25] and is an ongoing subject for discussion [6].

With growing computational resources and advances in the modeling of biomechanical material behavior, the simulation of patient-specific AAA models has been advanced by several research groups. Experiments on harvested AAA samples were able to reveal material parameters and failure properties. In addition with regression models [13, 26, 27] for the prediction of the individual wall strength, this enabled the definition of biomechanics-based indices [19, 20, 22, 28], such as the rupture potential index (RPI)

$$\begin{array}{c} \text{RPI}=\frac{\sigma_{\text{vm}}^{\text{max}}}{\sigma_{\gamma }}\;\;\;\text{or}\;\;\;\text{RPI}\left(x\right)=\frac{\sigma_{\text{vm}}\left(x\right)}{\sigma_{\gamma }\left(x\right)}, \end{array}$$ (5)

relating the von Mises stress to the wall strength. Furthermore, it could be shown [19, 20, 24] that these indices can be better rupture risk indicators than the maximum diameter criterion.

Experimental testing [13, 26, 27] also revealed significant inter- and intra-patient variabilities in the mechanical properties of AAA tissue, motivating a probabilistic approach to rupture risk estimation [14, 16, 21] and resulting in the probabilistic rupture risk index (PRRI) [21]

$$\begin{array}{c} \text{PRRI}=\int_{0}^{\infty }\int_{\sigma_{\gamma }}^{\infty }p\left(\sigma_{\text{vm}}^{\text{max}}\right)\;d\sigma_{\text{vm}}^{\text{max}}\;p\left(\sigma_{\gamma }\right)\;d\sigma_{\gamma }, \end{array}$$ (6)

where the authors used distributions for the wall thickness and wall strength that were fitted on cohort data published by our group [13].

#### 2.1.3 A novel probabilistic approach

**Fig 1:**
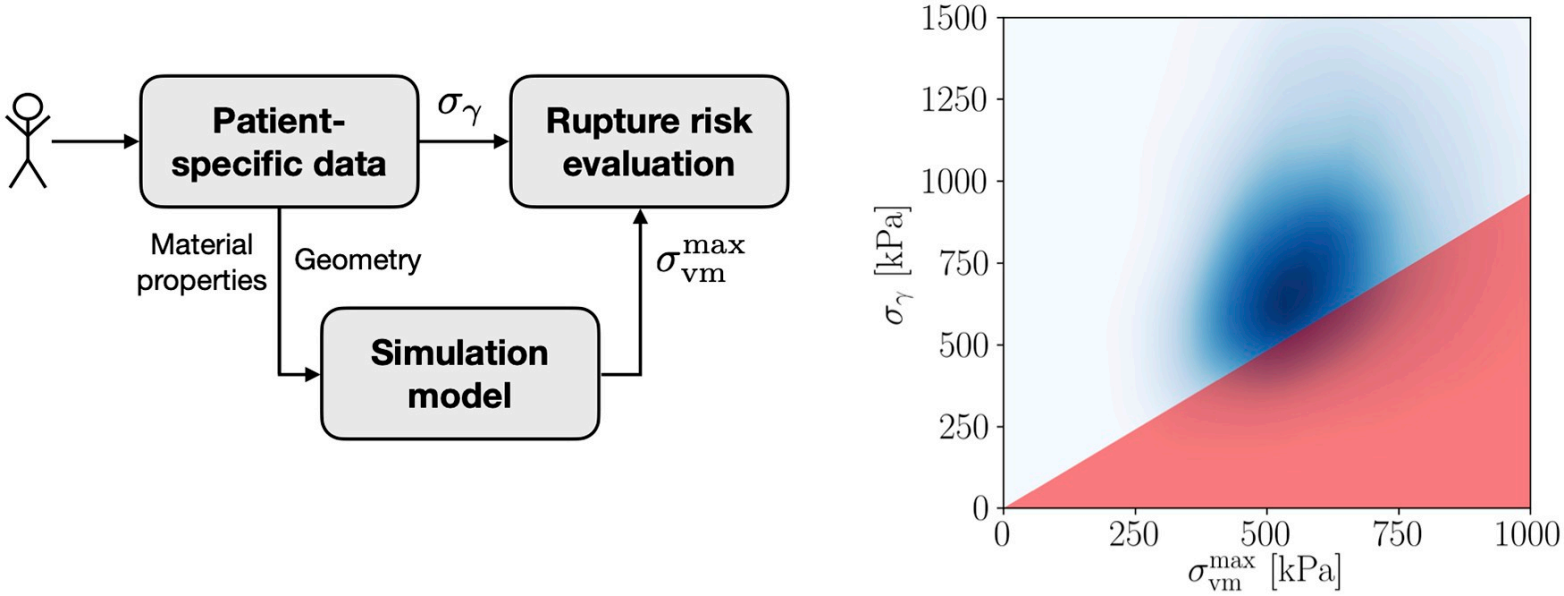
Rationale for our novel formulation (left) and exemplary visualization of its estimation (right). The probability of rupture, ${\mathbb{P}}_{rupt}$, is calculated as the volume of the probability distribution $p(\sigma_{vm}^{max},\sigma_{\gamma })$ within the triangular-shaped area marked in red.

In this work, we propose a novel failure-based, probabilistic rupture risk indicator that consistently incorporates all available statistical information and accounts for correlations among vessel wall properties. Fig 1 (left) illustrates the rationale for our approach, showing how part of the available data is directly involved in the estimation of the risk of rupture, while another part affects the evaluation of the computational model. In general, this data will be correlated, resulting in correlated quantities for the evaluation of rupture risk and necessitating a reformulation.

To that end and recalling the rupture criterion from Eq (3), we can calculate the probability of rupture over the joint probability distribution $p(\sigma_{vm}^{max},\sigma_{\gamma })$ as

$$\begin{array}{c} \begin{array}{c} P_{\text{rupt}}=E_{p(\sigma_{\text{vm}}^{\text{max}},\sigma_{\gamma })}[1_{\sigma_{\text{vm}}^{\text{max}}>\sigma_{\gamma }}]=\int_{0}^{\infty }\int_{0}^{\infty }1_{\sigma_{\text{vm}}^{\text{max}}>\sigma_{\gamma }}\;p\left(\sigma_{\text{vm}}^{\text{max}},\sigma_{\gamma }\right)\;d\sigma_{\text{vm}}^{\text{max}}\;d\sigma_{\gamma }, \end{array} \end{array}$$ (7)

where $1_{\sigma_{vm}^{max}>\sigma_{\gamma }}$ is the indicator function defined as

$$\begin{array}{c} 1_{\sigma_{\text{vm}}^{\text{max}}>\sigma_{\gamma }}=\{\begin{array}{cc} 1 & \sigma_{\text{vm}}^{\text{max}}>\sigma_{\gamma },\\ 0 & \text{otherwise}. \end{array} \end{array}$$ (8)

This formulation can be easily extended to, e.g., spatially varying vessel properties using Eqs (1) or (2) as failure events. Furthermore, it includes the PRRI in Eq (6) as a special case, when choosing $p(\sigma_{vm}^{max},\sigma_{\gamma })=p(\sigma_{vm}^{max})p(\sigma_{\gamma })$.

Lastly, it allows for a straightforward visual interpretation as illustrated in Fig 1 (right). The plot shows the joint probability distribution $p(\sigma_{vm}^{max},\sigma_{\gamma })$ and visualizes the rupture event area in red. The blue area implies a high probability for the joint occurrence of the corresponding stress and strength values. The probability of rupture ${\mathbb{P}}_{rupt}$ is simply the volume of this density within the triangular rupture event area. Thus, the larger the overlap between $p(\sigma_{vm}^{max},\sigma_{\gamma })$ and the red area, the higher ${\mathbb{P}}_{rupt}$.

### 2.2 Data-informed patient-specific AAA models

#### 2.1.1 Geometry creation from CT imaging and meshing

Patient-specific 3D AAA geometries are reconstructed via a semi-automatic segmentation process from CT imaging data using the software ScanIP (Synopsys, Mountain View, California) and based on a protocol as described in [12]. The minimal requirement for the spatial resolution of CT scans was 1 mm and for the slice thickness 3 mm. The upper boundary for the segmentation was the branching of the renal arteries and the lower boundary below the bifurcation at the iliac arteries. Due to the small thickness of the AAA wall, its low contrast and the limited resolution of the CT images, it is only possible to extract the blood lumen and intraluminal thrombus (ILT) geometries. After segmentation, the ILT geometry is exported as a surface model for meshing.

In a next step, we use the software Trelis (csimsoft, American Fork, Utah) and bi-linear quadrilateral elements to mesh the abluminal ILT surface. From this surface mesh, the arterial wall layer is extruded with a specified, spatially constant thickness *t*, resulting in a tri-linear, single layer, hexahedral mesh for the AAA wall. Finally, linear tetrahedral elements are employed for the meshing of the complex ILT geometry and a layer of linear pyramid elements as a transition for mesh compatibility between AAA wall and thrombus. Element sizes were set to 1.6 mm, corresponding to the median of measured thicknesses of AAA wall specimens in our database and leading to hexahedral elements of shape 1.6 mm × 1.6 mm × *t* for the AAA wall. A mesh convergence study has been performed to assess that the chosen spatial mesh resolution is sufficient in the context of our application. The meshing procedure is also described in [29] in more detail.

#### 2.2.2 Biomechanical modeling

Previous studies have shown that in order to accurately describe the biomechanical behavior of AAAs, a sufficient model complexity is required [20, 30], while results by [31, 32] indicate that also simpler models might be appropriate. For our purposes, we employ the finite deformation boundary value problem of nonlinear elasticity:

$$\begin{array}{cc} \nabla \cdot (FS)=0\;\;\; & \text{in}\;\;\;\Omega_{0}, \end{array}$$ (9)

$$\sigma \cdot n=\hat{t}\;\;\;\text{on}\;\;\;\gamma_{\sigma },$$ (10)

$$(FS)\cdot N=-k_{s}u\;\;\;\text{on}\;\;\;\Gamma_{u},$$ (11)

where *Ω*_0_ is the reference configuration of the AAA, **u** denotes the displacement field, **F** = **I**+ ∇**u** the deformation gradient, **S** the second Piola-Kirchhoff stress tensor and ***σ*** the Cauchy stress tensor.

On the Neumann boundary *γ*_*σ*_, i.e. the luminal ILT surface, an orthonormal load $\hat{t}=-pn$ is applied, with the pressure value *p* and the unit outward surface normal **n** in the current configuration. Furthermore, at the proximal and distal end surfaces of the AAA model, *Γ*_*u*_, we employ a Robin-type boundary condition with spring supports following [29, 33]. The stiffness parameter *k*_*s*_ is per unit reference area and set to 100 kPa/mm in this study, while **N** is the unit outward surface normal in the reference configuration.

To model the constitutive behavior of the ILT, we use the strain energy function proposed in [34]

$$\begin{array}{c} \Psi_{\text{ILT}}\left({\bar{I}}_{1},{\bar{I}}_{2},J\right)=c\left({\bar{I}}_{1}^{2}-2{\bar{I}}_{2}-3\right)+\Psi_{\text{vol}}\left(J\right) \end{array}$$ (12)

and a linearly decreasing stiffness *c* from the luminal to the abluminal ILT surface [12]. ${\bar{I}}_{1}$ and ${\bar{I}}_{2}$ are the first and second invariants of the modified right Cauchy-Green deformation tensor $\hat{C}=J^{-\frac{2}{3}}C$, with ***C*** = ***F***^T^ ***F*** and *J* = det(**F**) [29]. The strain energy function employed for the AAA wall material is [35, 36]

$$\begin{array}{c} \Psi_{\text{wall}}\left({\bar{I}}_{1},J\right)=\alpha ({\bar{I}}_{1}-3)+\beta {\left({\bar{I}}_{1}-3\right)}^{2}+\Psi_{\text{vol}}\left(J\right), \end{array}$$ (13)

with stiffness parameters *α* and *β*. Both strain energy functions are equipped with an additive volumetric component

$$\begin{array}{c} \Psi_{\text{vol}}\left(J\right)=\frac{\kappa }{4}(J^{2}-2\ln J-1), \end{array}$$ (14)

including the bulk modulus

$$\begin{array}{c} \kappa =\frac{{\bar{\kappa }}^{\left(\cdot \right)}}{1-2\nu } \end{array}$$ (15)

with parameters ${\bar{\kappa }}^{ILT}=8c$ and ${\bar{\kappa }}^{wall}=2\alpha$ for the employed ILT and wall material models and a Poisson’s ratio of *ν* = 0.48 [12].

To obtain a pressurized in vivo configuration of the AAA, the MULF prestressing method [10, 11] is used, where the applied load corresponds to the mean arterial pressure (MAP = 1/3 systolic pressure + 2/3 diastolic pressure). From this prestressed configuration, the pressure is raised by 50% to simulate elevated blood pressure conditions [21]. Following [19] and for comparability reasons, the values for the systolic and diastolic pressures were set to 121 mmHg and 87 mmHg for all cases, respectively, resulting in a MAP of 98.33 mmHg.

With the finite element discretization from Section 2.2.1, a nonlinear system of equations is obtained, which is solved using an in-house finite element code. We note that in this study we neglect the effect of calcifications in the AAA for simplicity and assume constant vessel wall thickness *t* and stiffness parameters *α* and *β* throughout the aneurysm. Furthermore, we evaluate the maximum von Mises stress as the 99*th* percentile of the von Mises stress field in the aneurysm.

For the remainder of this work, we will use the parameter to quantity of interest (QoI) map

$$\begin{array}{c} \sigma_{\text{vm}}^{\text{max}}=\sigma_{\text{vm}}^{\text{max}}\left(t,\alpha ,\beta \right)=\sigma_{\text{vm}}^{\text{max}}\left(\theta \right) \end{array}$$ (16)

with parameter vector $\theta ={\left[t,\alpha ,\beta \right]}^{T}\in R_{+}^{3}$ and QoI $\sigma_{vm}^{max}\in {\mathbb{R}}_{+}$ to denote the forward problem. Thus, calculating $\sigma_{vm}^{max}(\theta )$ for one realization of *t*, *α* and *β* will involve one evaluation of the nonlinear finite element model.

#### 2.2.3 Patient database

The modeling of patient-specific vessel wall properties here is based on data that has been collected during several research projects between 2008 and 2017 on the mechanobiological behavior of AAAs [13, 15]. The study was approved by the ethics committee of the University Hospital rechts der Isar, Technical University of Munich. AAA patients undergoing elective OSR (including emergency repair due to rupture) at the University Hospital rechts der Isar in Munich, Germany, were added to the database, whenever it was possible to extract tissue samples for mechanical testing. Apart from anamnesis and CT imaging data, hemograms were evaluated and one or more AAA tissue samples harvested during OSR. These samples were mechanically and histologically investigated, resulting in an exhaustive retrospective AAA database. Further information on data collection and experimental testing can be found in [13, 15]. To date, the database contains a total number of 305 entries from an equal number of tissue samples that were collected from 139 patients.

The data can be split into two groups. Invasive properties (cf. Table 1), denoted as $\Theta ={\left[t,\alpha ,\beta ,\sigma_{\gamma }\right]}^{T}\in R_{+}^{4}$, are properties, which have been determined retrospectively from AAA tissue samples and cannot be obtained for a prospective patient by using clinically established methods. They are, however, essential for the biomechanical modeling and simulation of AAAs and the calculation of the probability of rupture using Eq (7). Non-invasive properties (cf. Table 2), denoted by ***ξ***, on the other hand, can be determined with standard methods in the clinic. The subrenal diameter in Table 2 is measured directly below the renal arteries. If the aneurysm reached the renal arteries, the aortic diameter between the celiac artery and the superior mesenteric artery minus 2.5 mm was used instead [12].

**Table 1:** Invasive properties represent key vessel wall characteristics for a biomechanical rupture risk assessment.

*t*	Wall thickness	[mm]
*α*	Alpha stiffness	[kPa]
*β*	Beta stiffness	[kPa]
*σ*_*γ*_	Wall strength	[kPa]

They cannot be obtained prospectively by using clinically established methods and will be dealt with based on statistics from experimental testing of AAA tissue samples.

**Table 2:** Non-invasive properties overview.

General	Sex	m = 1, w = 0
	Age	y
	Symptomatic	yes = 1, no = 0
	Ruptured	yes = 1, no = 0
Geometry	Maximum AAA diameter	mm
	Maximum thrombus thickness	mm
	AAA length	mm
	Subrenal diameter	mm
Medication	Acetylsalicylic acid (ASA) / clopidogrel	yes = 1, no = 0
	Angiotensin-converting enzyme (ACE) inhibitors	yes = 1, no = 0
	Statins	yes = 1, no = 0
	Beta blockers	yes = 1, no = 0
	Antihypertensives	yes = 1, no = 0
	Diuretics	yes = 1, no = 0
	Oral hypoglycemic agents / insulin	yes = 1, no = 0
Anamnesis	Hypertension	yes = 1, no = 0
	Diabetes mellitus	yes = 1, no = 0
	Hyperlipidemia	yes = 1, no = 0
	Smoking status	yes = 1, no = 0
	Chronic kidney disease (CKD)	yes = 1, no = 0
	Coronary heart disease (CHD)	yes = 1, no = 0
	Peripheral vascular disease (PVD)	yes = 1, no = 0
Hemogram	Sodium	mmol/l
	Potassium	mmol/l
	Calcium	mmol/l
	High-sensitivity C-reactive protein (hsCRP)	mg/l
	Fibrinogen	mg/dl
	Urea	mg/dl
	Creatinine	mg/dl
	Creatine kinase	1/l
	Leukocytes	1,000/*μ*l
	Erythrocytes	Mio/*μ*l
	Thrombocytes	1,000/*μ*l
	Hemoglobin	g/dl
	Mean corpuscular hemoglobin (MCH)	pg/cell
	Mean corpuscular volume (MCV)	fl
	Mean corpuscular hemoglobin concentration (MCHC)	gHb/100ml

These can be determined with standard methods in the clinic and will be used as feature variables to predict the invasive properties of a prospective AAA patient.

Based on correlations between the invasive and non-invasive properties [13], the goal is to construct a statistical model for the patient-individualized prediction of vessel wall properties **Θ**(*ξ*) for a prospective new patient with non-invasive properties ***ξ***. While this process is described in Section 2.2.4, a preprocessing step for the dataset is essential, since values are missing both in the invasive and non-invasive properties for several cases in our database. Moreover, the relatively small number of available data, but high number of non-invasive properties, requires a feature selection process to identify the most important properties in ***ξ***. Similar to [15], we conduct the following preprocessing steps.

Non-invasive features in ***ξ***, where more than 30% of the data points had missing values and patients with more than 30% of missing features were excluded and all other missing non-invasive properties imputed with the corresponding median value across the population. As a consequence, the four parameters calcium, high-sensitivity C-reactive protein (hsCRP), creatine kinase and fibrinogen were disregarded. Afterwards, all non-invasive features were normalized.

Based on a correlation analysis using Spearman’s rank correlation coefficient (cf. S1 Table), the total number of features was reduced to a final selection of 8 variables: maximum AAA diameter, maximum thrombus thickness, AAA length, subrenal diameter, thrombocytes, hemoglobin, mean corpuscular hemoglobin (MCH), mean corpuscular volume (MCV). The restriction was done using a sequential forward selection algorithm similar to [15]. In an attempt to keep the number of non-invasive parameters small, we iteratively added the highest correlating non-invasive parameters to the GP model (see Section 2.2.4) until no further improvement in the leave-one-out cross-validation (LOOCV) scores could be observed. We note, however, that this does not imply that other non-invasive features such as sex, medication or anamnesis parameters do not have an influence on the biomechanical properties of the AAA wall. The resulting dataset $D=\{\xi_{i},\Theta_{i}{\}}_{i=1}^{n_{data}}$, that was used for the analysis in Section 3, consisted of *n*_data_ = 251 data points from 113 individual patients and is available as supplementary information to this study (cf. S2 and S3 Tables).

#### 2.2.4 Prediction of invasive vessel wall properties

Previous approaches to create models for the AAA wall thickness, stiffness parameters or strength were either deterministic [12, 26], based on cohort statistics [21], or did not account for correlations among the vessel wall quantities [15]. In the following, we make use of a multivariate Gaussian process regression model [37–39] to address these shortcomings and achieve the following desiderata:

1. Patient-specific modeling: obtain personalized estimates for the vessel wall quantities **Θ** based on correlations with the non-invasive properties ***ξ*** of a specific, prospective patient.
2. Probabilistic treatment: take into account the uncertainties in the predictions for **Θ** (do not ignore statistical information).
3. Dependencies: model the correlations among the invasive properties **Θ** in order to obtain a more accurate probabilistic description and avoid physically implausible parameter configurations.

As a result, given the non-invasive properties ***ξ*** of a prospective AAA patient, the logarithm (acting as a positivity constraint) of the corresponding prediction **Θ**(***ξ***) will follow a multivariate Gaussian distribution with predicted mean ***μ***_log**Θ**_ and covariance matrix Σ_log **Θ**_, i.e.

$$\begin{array}{c} \log\Theta \left(\xi \right)∼N(\mu_{\log\Theta },\Sigma_{\log\Theta })=p\left(\log\Theta \right). \end{array}$$ (17)

As we will see in Section 3.2, our approach leads to more accurate estimates for **Θ** and also a lower variance in the predictions. All relevant details regarding this model are provided in Appendix A.1.

### 2.3 A Kriging surrogate model for the maximum stress

#### 2.3.1 Estimating the probability of rupture

Since the calculation of the probability of rupture ${\mathbb{P}}_{rupt}$ from Eq (7) using the high-fidelity, nonlinear finite element model from Section 2.2.2 is infeasible for a clinical application, we propose a Kriging surrogate model to speed up computations [40–42]. The surrogate model will effectively serve as a proxy for the maximum von Mises stress $\sigma_{vm}^{max}(\theta )$ in the AAA vessel wall (cf. Eq (16)) and allows to make computationally cheap predictions at an arbitrary combination of ***θ*** = [*t*, *α*, *β*]^T^, i.e.

$$\begin{array}{c} \log\sigma_{\text{vm}}^{\text{max}}\left(\theta \right)∼N(\mu_{\log\sigma_{\text{vm}}^{\text{max}}},\delta_{\log\sigma_{\text{vm}}^{\text{max}}}^{2}), \end{array}$$ (18)

with the predicted mean $\mu_{\text{log}\sigma_{vm}^{max}}$ and standard deviation $\delta_{\text{log}\sigma_{vm}^{max}}$, respectively. For all relevant details, we refer to Appendix A.2. The high-fidelity model can then be simply approximated as $\text{log}\sigma_{vm}^{max}(\theta )\approx \mu_{\text{log}\sigma_{vm}^{max}}(\theta )$, allowing for a direct Monte Carlo estimation of the probability of rupture

$$\begin{array}{c} \begin{array}{c} P_{\text{rupt}}=E_{p(\log\Theta )}[1_{\log\sigma_{\text{vm}}^{\text{max}}\left(\theta \right)>\log\sigma_{\gamma }}]\approx \frac{1}{n_{\text{eval}}}\sum_{i=1}^{n_{\text{eval}}}1_{\log\sigma_{\text{vm}}^{\text{max}}\left(\theta_{i}\right)>\log\sigma_{\gamma ,i}}, \end{array} \end{array}$$ (19)

where

$$\begin{array}{c} 1_{\log\sigma_{\text{vm}}^{\text{max}}\left(\theta_{i}\right)>\log\sigma_{\gamma ,i}}=\{\begin{array}{cc} 1 & \log\sigma_{\text{vm}}^{\text{max}}\left(\theta_{i}\right)>\log\sigma_{\gamma ,i},\\ 0 & \text{otherwise} \end{array} \end{array}$$ (20)

and **Θ**_*i*_ ∼ *p*(log **Θ**), *i* = 1…*n*_eval_.

#### 2.3.2 An active learning approach to training

The Kriging surrogate training process is carried out under the following two demands:

1. As few as possible high-fidelity model evaluations.
2. Ensure that the Kriging model is accurate where necessary.

To that end, we adopt and extend the Active Learning-MacKay (ALM) strategy from [43] and choose points for high-fidelity model evaluations such as to minimize a density- and stress-weighted predictive standard deviation objective function

$$\begin{array}{c} \psi \left(\Theta \right)=\delta_{\log\sigma_{\text{vm}}^{\text{max}}}\left(\theta \right)\;p\left(\log\Theta \right)\;\mu_{\log\sigma_{\text{vm}}^{\text{max}}}\left(\theta \right), \end{array}$$ (21)

where *p*(log **Θ**) is the patient-specific probability distribution for the invasive model parameters **Θ** = [*t*, *α*, *β*, *σ*_*γ*_]^T^ from the regression model in Section 2.2.4. The reasoning behind this choice follows from the ALM approach, where only the predictive standard deviations $\delta_{\text{log}\sigma_{vm}^{max}}(\theta )$ are considered in the objective function. In our case, we are equipped with a probability distribution, *p*(log **Θ**), so we can attribute a higher weight to the more probable regions in **Θ**. Additionally, we pay special attention to points in the input space, where the predicted maximum von Mises stresses $\mu_{\text{log}\sigma_{vm}^{max}}$ are high to ensure the surrogate model accurately replicates the full model in these regions. The problem of choosing an appropriate point *θ*_next_ for evaluation results in the optimization problem

$$\begin{array}{c} \Theta_{\text{next}}=\arg\max_{\Theta }\;\psi \left(\Theta \right), \end{array}$$ (22)

which is approximated by creating a grid $\{\Theta_{i}{\}}_{i=1}^{n_{grid}}$ over the input space, calculating $\{\psi (\Theta_{i}){\}}_{i=1}^{n_{grid}}$ using the Kriging surrogate and determining

$$\begin{array}{c} \Theta_{\text{next}}\approx \arg\max_{\Theta }\;{\left\{\psi \left(\Theta_{i}\right)\right\}}_{i=1}^{n_{\text{grid}}}. \end{array}$$ (23)

The next evaluation point *θ*_next_ = [*t*_next_, *α*_next_, *β*_next_]^T^ can then simply be extracted from **Θ**_next_. During the active learning, we monitor the average

$$\begin{array}{c} \hat{\psi }=\frac{1}{n_{\text{grid}}}\sum_{i=1}^{n_{\text{grid}}}\psi \left(\Theta_{i}\right) \end{array}$$ (24)

and stop the training process, when there are no more significant changes in $\hat{\psi }$ with an increasing number of high-fidelity model evaluations.

## 3 Results

### 3.1 Framework summary

Based on our retrospective AAA database of non-invasive and invasive data pairs and a multi-output Gaussian process model fitted to this dataset (cf. Section 2.2.4), the necessary steps to estimate the probability of rupture for a prospective patient are:

- **Step 1:** Data generation in the clinic: CT imaging, determination of the non-invasive parameters ***ξ*** from Table 2
- **Step 2:** Geometry creation: segmentation and meshing of the AAA geometry
- **Step 3:** Model specification: modeling of the invasive properties **Θ**(***ξ***) using the multi-output Gaussian process model from Sections 2.2.4 and A.1.
- **Step 4:** Surrogate training: fitting of the Kriging model using active learning
- **Step 5:** Post-processing: estimating the probability of rupture

While CT imaging is essential for geometry creation, the rupture risk analysis can also be carried out if no non-invasive properties ***ξ*** are available for a prospective patient by using cohort statistics (cf. Model 1, Section 3.2) without personalization. The computational procedure is summarized in Algorithm 1. In practice, it has proven feasible to choose *n*_init_ = 8 (where it makes sense to include the predicted mean ***μ***_log **Θ**_ in the set of initial samples), *n*_grid_ = *n*_eval_ = 10, 000 and tol = 1.0 × 10^−4^.

**Algorithm 1** Calculating the probability of rupture ${\mathbb{P}}_{rupt}$

1: **Input:** Input uncertainties *p*(log**Θ**(***ξ***)), simulation model $\sigma_{vm}^{max}(\theta )$, tol, *n*_init_, *n*_grid_, *n*_eval_

2: Set $iter=1,{\hat{\psi }}_{0}=0$

3: Generate *n*_init_ samples $\{\text{log}\theta_{i}{\}}_{i=1}^{n_{init}}$ and calculate $\{\text{log}\sigma_{vm}^{max}(\theta_{i}){\}}_{i=1}^{n_{init}}$

4: Train a Kriging surrogate using the training data $\{\theta_{i},\text{log}\sigma_{vm}^{max}(\theta_{i}){\}}_{i=1}^{n_{init}}$

5: Create a grid $\{\text{log}\Theta_{i}{\}}_{i=1}^{n_{grid}}$ over the input space and calculate ${\hat{\psi }}_{1}$ (cf. Eq (24))

6: **while** $|{\hat{\psi }}_{iter}-{\hat{\psi }}_{iter-1}|>tol$ **do**

7:  Determine *θ*_next_ using Eq (23) and calculate $\sigma_{vm}^{max}(\theta_{next})$

8:   Update the Kriging model with the new data point $\left\{\theta_{next},\text{log}\sigma_{vm}^{max}(\theta_{next})\right\}$ and calculate ${\hat{\psi }}_{iter}$

9:  Set iter = iter + 1

10: **end while**

11: Generate *n*_eval_ samples $\{\text{log}\Theta_{i}{\}}_{i=1}^{n_{eval}}$ and calculate ${\mathbb{P}}_{rupt}$ according to Eq (19) using the Kriging surrogate

12: **Output:** ${\mathbb{P}}_{rupt}$

### 3.2 Regression model benchmark

Before demonstrating the framework in full detail, a brief comparison between the multi-output Gaussian process regression model (cf. Section 2.2.4) with existing probabilistic modeling approaches used in the context of AAA rupture risk is provided. To that end, we employ leave-one-out-cross-validation (LOOCV) on our dataset D (cf. Section 2.2.3) to test the predictive capabilities of three different models for *p*(log**Θ**):

- Model 1: assuming all variables are log-normally distributed and independent, the joint distribution
  $$\begin{array}{c} p\left(\log\Theta \right)=N\left(\mu_{\log t},\sigma_{\log t}^{2}\right)N\left(\mu_{\log\alpha },\sigma_{\log\alpha }^{2}\right)N\left(\mu_{\log\beta },\sigma_{\log\beta }^{2}\right)N\left(\mu_{\log\sigma_{\gamma }},\sigma_{\log\sigma_{\gamma }}^{2}\right) \end{array}$$ (25)
  is obtained, where the means and variances are calculated across the whole population using the dataset D, that is
  $$\begin{array}{c} \mu_{\log\kappa }=\frac{1}{n_{\text{data}}}\sum_{i=1}^{n_{\text{data}}}\log\kappa_{i}\;\;\;\text{and}\;\;\;\sigma_{\log\kappa }^{2}=\frac{1}{n_{\text{data}}}\sum_{i=1}^{n_{\text{data}}}{\left(\log\kappa_{i}-\mu_{\log\kappa }\right)}^{2}, \end{array}$$ (26)
  with *κ* ∈ {*t*, *α*, *β*, *σ*_*γ*_}. This corresponds to the approach chosen in [21].
- Model 2: by training single-output Gaussian processes for each output variable separately following [15], the same decomposition of Gaussian distributions as in Eq (25) is obtained, however, with means and variances predicted individually for each patient.
- Model 3: our proposed multi-output Gaussian process (cf. Eq (17)).

In addition to the mean of the patient standardized mean square error (PSMSE) [15], we also report the mean of the patient predictive entropy (PPE),

$$\begin{array}{c} E\left[\text{PPE}\right]=\frac{1}{n_{\text{pat}}}\sum_{i=1}^{n_{\text{pat}}}H\left[p_{i}\left(\log\Theta \right)\right], \end{array}$$ (27)

where ℍ[p(logΘ)] is the entropy of the distribution *p*(log**Θ**) and a measure of uncertainty or variance for multivariate distributions. With regards to the different measures, it is desirable for both PSMSE and PPE to be small, corresponding to a model which is accurate and produces low-variance estimates. For conciseness, values for the mean of the PSMSE are averaged over the four predictive variables **Θ**. We refer to [15] for an exhaustive discussion of the LOOCV and calculation of the PSMSE. The obtained results for the three models are shown in Table 3. We note that our proposed model (Model 3) was able to consistently achieve the lowest scores, although the differences are rather small.

**Table 3:** Leave-one-out-cross-validation (LOOCV) results for the three probabilistic models.

	Model 1	Model 2	Model 3
E[PSMSE]	0.9480	0.9315	0.9226
E[PPE]	3.5778	3.4300	3.3353

The table compares the calculated mean (E[PSMSE]) of the patient standardized mean square error (PSMSE) averaged over the four predictive variables **Θ** as well as the mean of the patient predictive entropy (E[PPE]).

### 3.3 Framework demonstration for AAA Pat17

To illustrate the application of our proposed framework we demonstrate all steps in detail below, following the outline as presented in Section 3.1. We assume we are provided with CT imaging data and non-invasive properties ***ξ*** for one specific prospective AAA (**Step 1**), referred to as Pat17 in the following.

**Fig 2:**
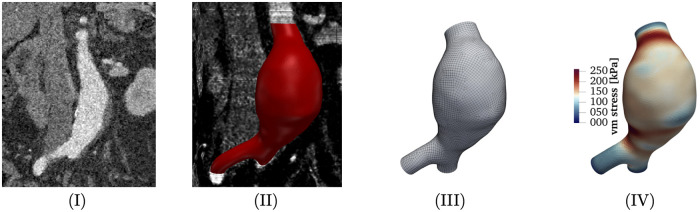
AAA Pat17 as seen via CT imaging (I), a 3D rendering of the segmentation result (II), the generated finite element mesh (III) and a visualization of the von Mises stress field corresponding to the mean *μ*_logΘ_ of the predictive distribution *p*(logΘ) for that AAA (IV).

Fig 2 shows the AAA as seen via CT imaging (I), a 3D rendering of the segmentation result (II) as well as the generated finite element mesh (III) (**Step 2**). The mesh consists of 117, 218 finite elements and 93, 840 nodal degrees of freedom, with an approximate element size of 1.6 mm.

**Fig 3:**
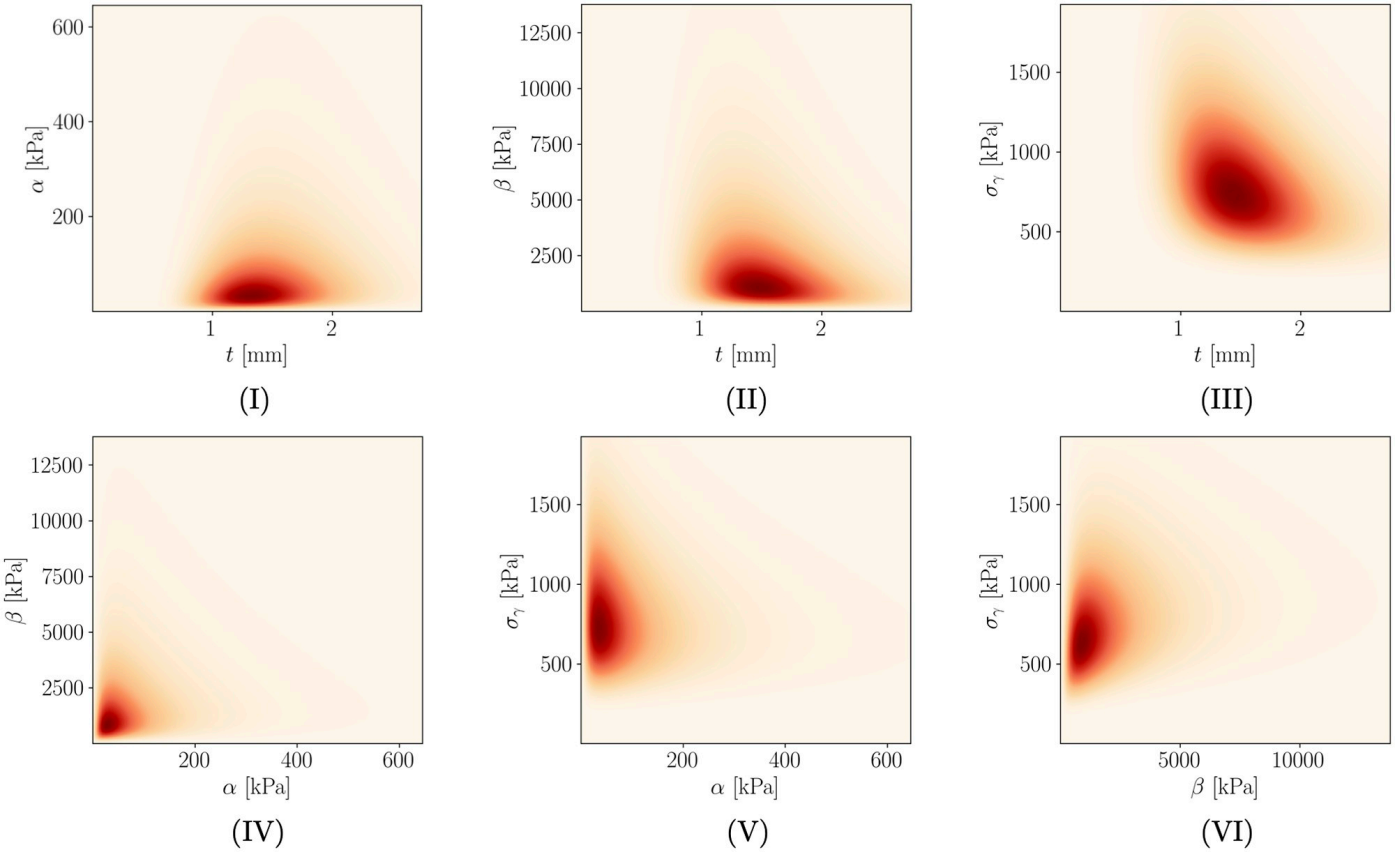
Visualization of the predictive distribution *p*(logΘ) transformed to the physical parameter range for AAA Pat17. Plots (I)-(VI) show 2D marginal distributions over all possible parameter combinations between *t*, *α*, *β* and *σ*_*γ*_. Highest correlations are observed between *t* and *σ*_*γ*_ ($\rho_{t,\sigma_{\gamma }}=-0.3214$), *β* and *σ*_*γ*_ ($\rho_{\beta ,\sigma_{\gamma }}=0.2274$), *t* and *β* (*ρ*_*t*,*β*_ = −0.1966) as well as *α* and *β* (*ρ*_*α*,*β*_ = 0.1413).

Table 4 shows the relevant 8 non-invasive properties ***ξ*** that are used by the regression model (cf. Section 2.2.4) to obtain the predictive distribution *p*(log**Θ**(***ξ***)), which is specific to Pat17. Along with that, means and standard deviations based on all 113 patients in D are provided. Based on this data, we can predict the mean ***μ***_log**Θ**_ and covariance *Σ*_log**Θ**_ for this patient (**Step 3**). The obtained distribution is visualized in Fig 3 and the predictive means and standard deviations are provided in Table 5 along with reference values from the cohort. The entropy of *p*(log**Θ**) is 3.3050 and thus slightly lower than the LOOCV mean (cf. Table 3). Highest correlations among the invasive properties for Pat17 can be found between *t* and *σ*_*γ*_ ($\rho_{t,\sigma_{\gamma }}=-0.3214$), *β* and *σ*_*γ*_ ($\rho_{\beta ,\sigma_{\gamma }}=0.2274$), *t* and *β* (*ρ*_*t*,*β*_ = −0.1966) as well as *α* and *β* (*ρ*_*α*,*β*_ = 0.1413).

**Table 4:** Non-invasive properties *ξ* for AAA Pat17 as well as cohort means and standard deviations (based on all 113 patients in D) for comparison.

		Pat17	Cohort (mean±std)
Maximum AAA diameter	[mm]	53.75	62.91±17.57
Subrenal diameter	[mm]	21.88	24.58±6.55
AAA length	[mm]	85.0	111.84±28.30
Maximum thrombus thickness	[mm]	19.11	24.10±11.19
Thrombocytes	[1,000/*μ*l]	182.0	221.33±82.10
Hemoglobin	[g/dl]	15.1	13.27±2.20
Mean corpuscular hemoglobin (MCH)	[pg/cell]	29.0	30.39±2.46
Mean corpuscular volume (MCV)	[fl]	85.0	89.95±6.61

**Table 5:** Predicted means and standard deviations for the invasive properties of AAA Pat17 along with cohort values over all *n*_data_ = 251 samples for comparison.

		Pat17 (mean±std)	Cohort (mean ± std)
log*t*		0.415 ± 0.088	0.484 ± 0.105
*t*	[mm]	1.583 ± 0.481	1.710 ± 0.568
log*α*		4.504 ± 0.967	4.543 ± 1.036
*α*	[kPa]	146.529 ± 187.106	157.676 ± 212.579
log*β*		7.723 ± 0.817	7.685 ± 0.758
*β*	[kPa]	3399.204 ± 3811.469	3178.355 ± 3383.842
log*σ*_*γ*_		6.729 ± 0.174	6.704 ± 0.183
*σ*_*γ*_	[kPa]	912.004 ± 397.176	894.182 ± 400.798

**Fig 4:**
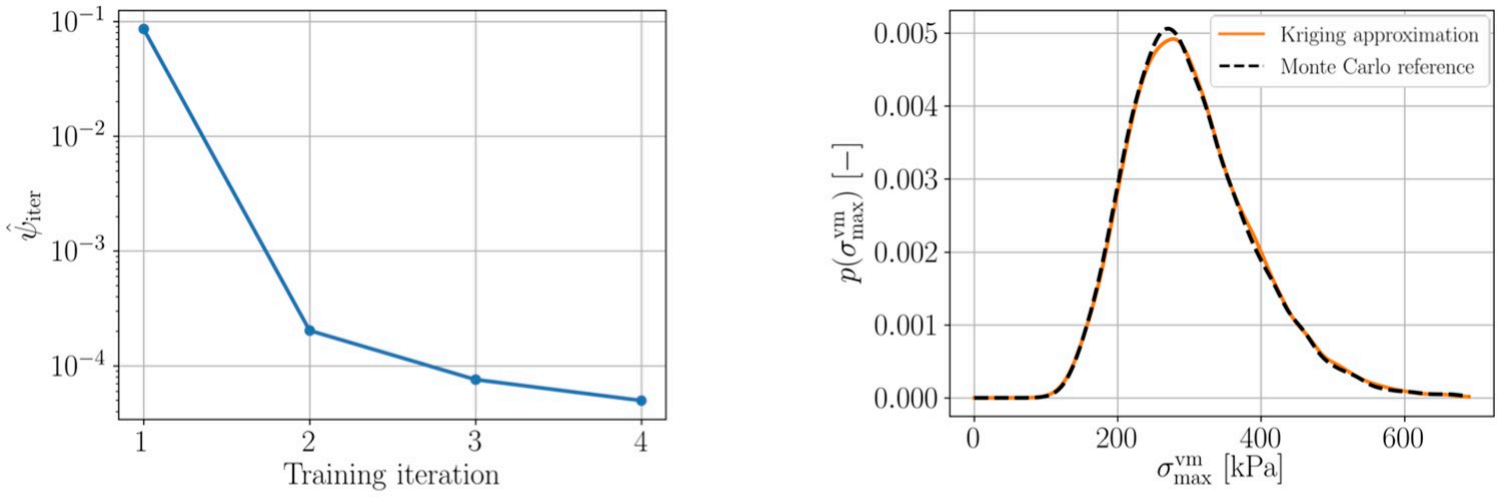
Left: Decrease of the objective function over the number of training iterations, where the first training iteration corresponds to the Kriging surrogate after *n*_init_ = 8 model evaluations. 11 model evaluations were used for the surrogate creation. Right: Estimated Kriging-based distribution $p(\sigma_{max}^{vm})$ along with a Monte Carlo reference. All densities were calculated using kernel density estimation with Gaussian kernels based on 10, 000 samples of the maximum von Mises stress $\sigma_{max}^{vm}$.

Given *p*(log**Θ**), the forward model (cf. Eq (16)) for Pat17 is defined. The probability of rupture for this AAA is approximated using a Kriging surrogate model (**Step 4**). Fig 2 (IV) provides a visualization of the von Mises stresses corresponding to ***μ***_log**Θ**_, the mean parameter combination of *p*(log**Θ**). Fig 4 shows the decrease of the objective function over the number of iterations on the left as well as a comparison of the Kriging-based approximate distribution $p(\sigma_{vm}^{max})$ together with a Monte Carlo reference calculated using 10, 000 samples on the right.

**Fig 5:**
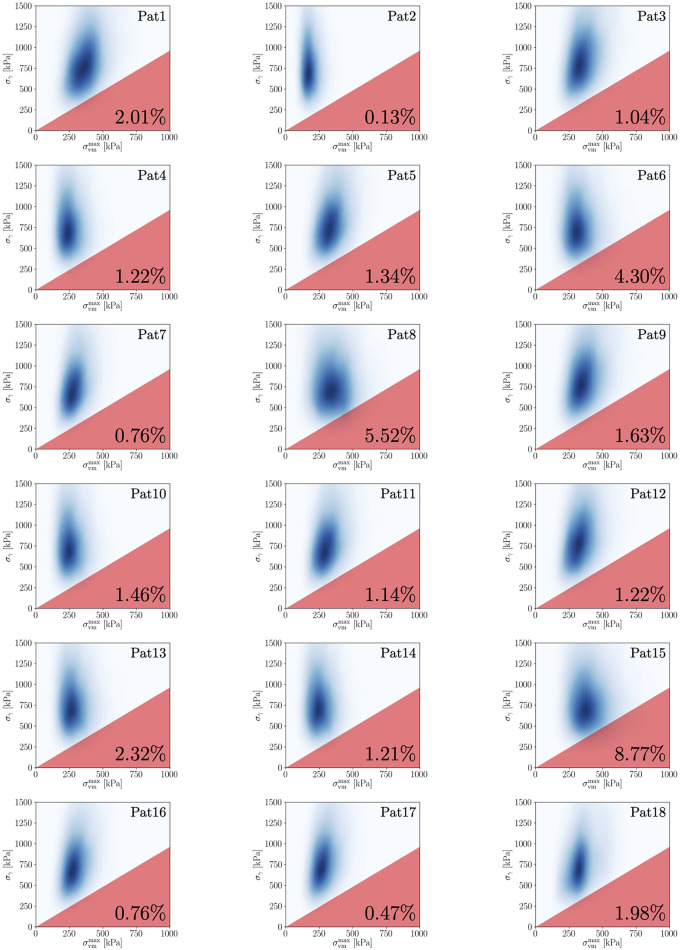
Visualization of ${\mathbb{P}}_{rupt}$ for all AAAs in group 1.

Lastly, the probability of rupture can be estimated using the Kriging surrogate (**Step 5**), which amounts to 0.47% for Pat17 (cf. Fig 5 for a visualization). We stress that this value must not be compared to the operative risks associated with OSR or EVAR in order to make decisions. Rather, it needs to be put into context with results for other AAA patients that have been computed using the same methodology, which is discussed below in Section 3.4.

### 3.4 Comparative case-control study using diameter matched groups

To test the efficacy of the framework as a rupture risk indicator and to compare it with existing biomechanical indices, we consider diameter matched groups of asymptomatic (group 1, *n* = 18) and known symptomatic/ruptured (group 2, *n* = 18) AAA patients from our database. The groups were chosen such that their maximum diameter mean and standard deviation approximately match (group 1: 62.17±7.18 mm, group 2: 63.06±7.56 mm), rendering a differentiation between the groups based on the maximum diameter criterion ineffective.

For a detailed overview regarding the selection of the two groups, we refer to Table 6. After preprocessing of our original dataset (cf. Section 2.2.3), we restricted the cohort to AAAs with a maximum diameter between 50 and 80 mm in order to obtain an intermediate-sized group of patients. As a result, 64 patients remained, of which 47 had asymptomatic and 17 had symptomatic or ruptured AAAs. The latter were put into one group, since symptomatic AAAs are known to be at an elevated risk of rupture [44]. The reason for the much lower number of symptomatic/ruptured AAAs is that these AAAs often have very large diameters (>80mm). We included AAA patients from a previous case-control study by our group [19], which examined 13 asymptomatic and 12 symptomatic AAA patients. Finally, we manually selected 18 asymptomatic and 18 symptomatic/ruptured patients based on the following criteria:

- Find two groups with the best match in diameter.
- Preferably include cases where non-invasive data is available and thus patient-specific invasive properties can be predicted.
- Disregard cases, where CT images are not available or lack a sufficient image quality to create simulation models.

**Table 6:** Overview: Selection process for the diameter matched groups.

	total no.	♂	♀	asympt	sympt/rupt
original database	139	122	17	100	39
after preprocessing	113	99	14	83	30
diameter filter	64	58	6	47	17
manual selection	19	19	0	10	9
added from [19]	17	12	5	8	9
final cohort	36	31	5	18	18

**Fig 6:**
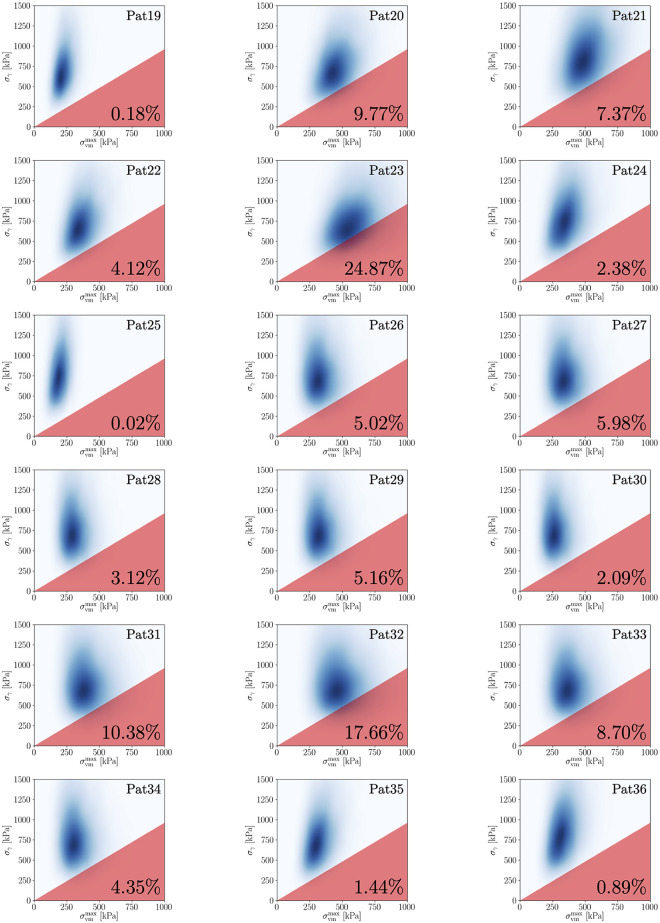
Visualization of ${\mathbb{P}}_{rupt}$ for all AAAs in group 2.

Detailed information for all AAAs of both groups is provided in Tables 7 and 8 and a visualization of their rupture risk indices, ${\mathbb{P}}_{rupt}$, in Figs 5 and 6 (cf. Appendix A.3). No patient had known connective tissue disorders. For 10 out of 18 AAAs in group 1 and for 9 out of 18 AAAs in group 2 we had non-invasive data and were thus able to use the multi-output regression model to determine a personalized input density *p*(log**Θ**). For the remaining 8 (group 1) and 9 (group 2) AAAs, we used cohort statistics, i.e. Model 1 from Section 3.2.

**Table 7:** Group 1 (asymptomatic, 18 ♂, 0 ♀) overview and obtained results for $\sigma_{max}^{vm}$, RPI, PRRI and ${\mathbb{P}}_{rupt}$.

Nr.	*d*_max_ [mm]	$\sigma_{max}^{vm}$ [kPa]	RPI [−]	PRRI [%]	${\mathbb{P}}_{rupt}$ [%]
Pat1	63.09	373.14	0.398	6.48	2.01
Pat2	69.23	180.21	0.202	0.20	0.13
Pat3	61.76	368.65	0.362	4.20	1.04
Pat4	50.37	257.04	0.288	1.55	1.22
Pat5	62.94	349.00	0.371	4.15	1.34
Pat6	61.10	324.35	0.363	3.81	4.30
Pat7	54.94	301.55	0.339	3.06	0.76
Pat8	60.14	348.62	0.390	5.36	5.52
Pat9	57.12	380.97	0.382	5.68	1.63
Pat10	57.94	263.15	0.295	1.65	1.46
Pat11	57.63	324.06	0.359	3.93	1.14
Pat12	55.35	343.26	0.356	3.84	1.22
Pat13	66.25	281.44	0.315	2.14	2.32
Pat14	71.25	255.60	0.286	1.49	1.21
Pat15	70.52	394.89	0.442	8.32	8.77
Pat16	79.94	300.20	0.342	4.06	0.76
Pat17	53.75	291.70	0.320	1.93	0.47
Pat18	65.81	344.30	0.393	5.37	1.98
mean	62.17	315.67	0.345	3.73	2.07
std	7.18	53.18	0.053	1.99	2.07
25th percentile	57.25	284.00	0.316	1.98	1.07
50th percentile	61.43	324.21	0.357	3.89	1.28
75th percentile	66.14	348.90	0.379	5.07	2.00

**Table 8:** Group 2 (symptomatic/ruptured, 13♂, 5 ♀) overview and obtained results for $\sigma_{max}^{vm}$, RPI, PRRI and ${\mathbb{P}}_{rupt}$.

Nr.	*d*_max_ [mm]	$\sigma_{max}^{vm}$ [kPa]	RPI [−]	PRRI [%]	${\mathbb{P}}_{rupt}$ [%]
Pat19	57.55	230.60	0.282	1.08	0.18
Pat20	70.40	473.52	0.551	16.38	9.77
Pat21	70.76	538.30	0.507	15.16	7.37
Pat22	73.32	380.57	0.452	9.47	4.12
Pat23	77.09	738.58	0.860	30.03	24.87
Pat24	72.80	377.91	0.404	6.51	2.38
Pat25	52.26	197.94	0.220	0.25	0.02
Pat26	60.95	335.47	0.376	4.92	5.02
Pat27	60.30	359.65	0.403	6.21	5.98
Pat28	53.75	309.83	0.347	3.23	3.12
Pat29	55.69	340.56	0.381	4.90	5.16
Pat30	53.53	281.85	0.316	2.47	2.09
Pat31	60.93	412.86	0.462	10.33	10.38
Pat32	70.52	495.17	0.555	17.27	17.66
Pat33	67.10	393.87	0.441	8.40	8.70
Pat34	56.59	328.43	0.368	4.21	4.35
Pat35	60.58	329.85	0.369	4.41	1.44
Pat36	60.93	341.59	0.346	4.11	0.89
mean	63.06	381.47	0.424	8.30	6.31
std	7.56	119.61	0.135	7.18	6.21
25th percentile	56.83	328.78	0.352	4.14	2.16
50th percentile	60.93	350.62	0.392	5.57	4.69
75th percentile	70.49	408.12	0.460	10.12	8.37

We apply our framework to all 36 AAAs using an individual prospective scenario, i.e. before starting the analysis for one AAA, this patient is removed from the database, while the other 35 AAAs are included. In order to provide a comparison of ${\mathbb{P}}_{rupt}$ with other biomechanical indices, we calculate the following additional quantities:

- Maximum von Mises stress at the input parameter mean (neglects any statistical information):
  $$\begin{array}{c} \sigma_{\text{max}}^{\text{vm}}\left(\mu_{\log\theta }\right). \end{array}$$ (28)
- Rupture potential index [28] at the input parameter mean (neglects any statistical information, but takes into account the wall strength):
  $$\begin{array}{c} \text{RPI}=\frac{\sigma_{\text{max}}^{\text{vm}}\left(\mu_{\log\theta }\right)}{\mu_{\sigma_{\gamma }}}. \end{array}$$ (29)
- Probabilistic rupture risk index [21] (takes into account cohort-based uncertainties in the wall thickness and wall strength according to Model 1, Section 3.2):
  $$\begin{array}{c} \text{PRRI}=E_{N\left(\mu_{\log t},\sigma_{\log t}^{2}\right)N\left(\mu_{\log\sigma_{\gamma }},\sigma_{\log\sigma_{\gamma }}^{2}\right)}[1_{\log\sigma_{\text{vm}}^{\text{max}}\left(\mu_{\log t}\right)>\mu_{\log\sigma_{\gamma }}}]. \end{array}$$ (30)

**Fig 7:**
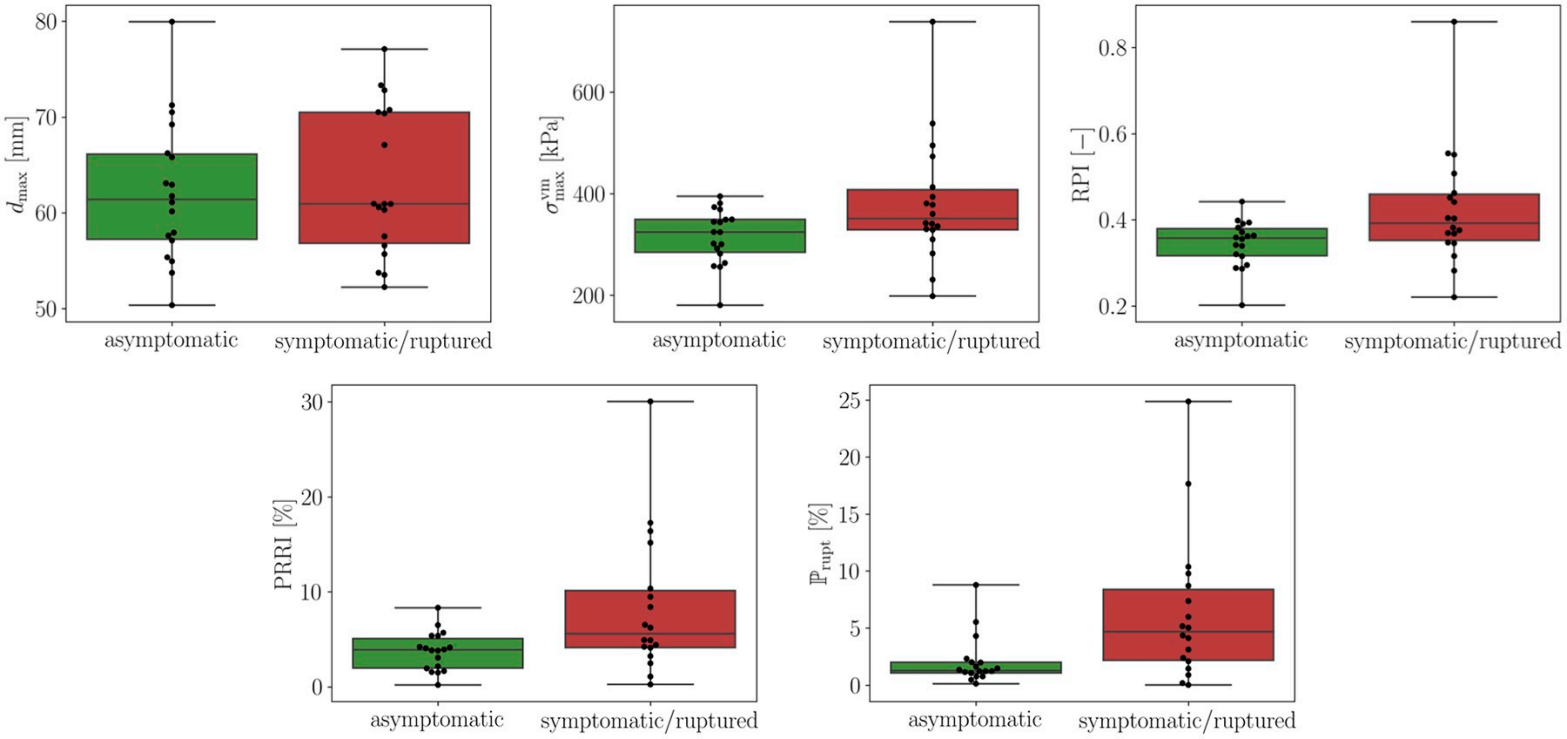
Boxplots comparing *d*_max_, $\sigma_{max}^{vm}$, RPI, PRRI and ${\mathbb{P}}_{rupt}$ for the asymptomatic and symptomatic/ruptured group. The plots illustrate the interquartile range (green and red color) including the sample median as well as the first and third quartiles. Whiskers indicate minimum and maximum values and black dots represent all values from the respective group.

**Fig 8:**
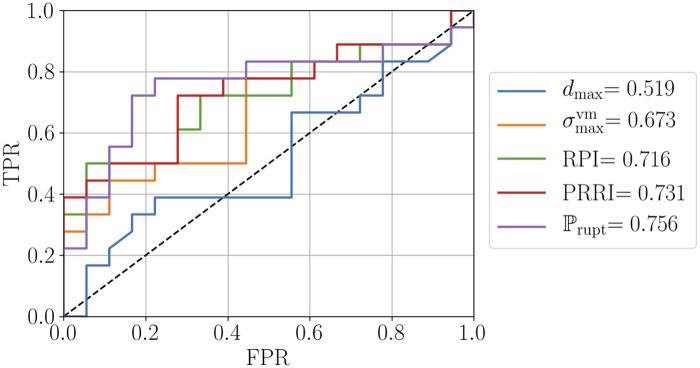
Receiver operating characteristic (ROC) curves showing true positive rates (TPR) over false positive rates (FPR) and area under the ROC curve (AUC) scores for *d*_max_, $\sigma_{max}^{vm}$, RPI, PRRI and ${\mathbb{P}}_{rupt}$.

Comprehensive results for all patients are listed in Tables 7 and 8 (cf. Appendix A.3). The average number of high-fidelity model evaluations to train the Kriging surrogate was 11. Based on these results and to evaluate the performance of the individual quantities, we provide:

1. Relative mean and median differences between group 1 and group 2 (cf. Table 9).
2. Boxplots for both groups (cf. Fig 7).
3. Receiver operating characteristic (ROC) curves and the area under the ROC curve (AUC) (cf. Fig 8) [45]. Computed true positive rates (TPR), false positive rates (FPR) and corresponding threshold values are provided for ${\mathbb{P}}_{rupt}$ as supplementary information (cf. S4 Table).

**Table 9:** Relative mean and median differences (in %) of *d*_max_, $\sigma_{max}^{vm}$, RPI, PRRI and ${\mathbb{P}}_{rupt}$ between the asymptomatic and the symptomatic/ruptured group.

		*d*_max_	$\sigma_{max}^{vm}$	RPI	PRRI	${\mathbb{P}}_{rupt}$
Δ mean	[%]	1.42	20.84	23.15	122.17	204.45
Δ median	[%]	0.81	8.15	9.75	43.24	266.02

Relative differences for a quantity *q* between the asymptomatic group result *q*_a_ and the symptomatic/ruptured group result *q*_s/r_ are calculated as Δ*q* = |*q*_s/r_ − *q*_a_|/*q*_a_.

## 4 Discussion

The obtained values for the relative mean and median differences in Table 9 confirm that group 1 and group 2 are indistinguishable based on the maximum diameter criterion. While the relative differences are higher for $\sigma_{max}^{vm}$ and RPI, PRRI and in particular our proposed index ${\mathbb{P}}_{rupt}$ feature a significantly larger mean and median difference. Recalling that the maximum diameter, *d*_max_, is one important non-invasive parameter in our framework (cf. Section 2.2.3), we emphasize that its influence has been rendered ineffective through the study design. A similar trend as in Table 9 can be observed in Fig 7, with RPI and PRRI providing a slightly better separation between the two groups than $\sigma_{max}^{vm}$, while for ${\mathbb{P}}_{rupt}$ the interquartile ranges of the two groups are non-overlapping. Finally, in Fig 8 we can observe that ${\mathbb{P}}_{rupt}$ outperforms the remaining classifiers and achieves the best performance among all quantities in terms of the AUC score, followed by PRRI, RPI and $\sigma_{max}^{vm}$. We further note that from the 18 patients in the symptomatic/ruptured group, 11 had ruptured AAAs (Pat19, Pat23, Pat24, Pat26, Pat27, Pat28, Pat29, Pat30, Pat32, Pat34, Pat35). The mean ${\mathbb{P}}_{rupt}$ scores for the 11 ruptured AAAs is 6.57 and thus slightly higher than the mean 5.89 for the 7 symptomatic AAAs. To summarize our key observations:

- The maximum diameter criterion, by design, clearly fails to separate the two groups in all our comparisons.
- The proposed index ${\mathbb{P}}_{rupt}$ consistently achieves the best separation.
- The results indicate that the more statistical information taken into account, the better the capability to distinguish between group 1 and group 2.

Before translating these findings into any clinical application, however, there are several limitations that have to be kept in mind. First, this is a non-randomized, retrospective case-control study with a relatively small cohort size (group 1: *n* = 18, group 2: *n* = 18) and the database described in Section 2.2.3. Second, there was no matching based on other risk factors such as sex, age or family history, which could be a confounder. Third, since we only have access to electively repaired or symptomatic/ruptured AAAs for mechanical testing, the mean diameters of the two groups (group 1: 62.17 mm, group 2: 63.03 mm) are larger than the Society for Vascular Surgery’s decision criterion for elective repair (55 mm) [1]. In the future, due to the increasing use of EVAR, it will be even harder to obtain representative tissue samples from AAAs of relevant size for a database. As a result, caution is advised when interpreting the results presented here for smaller AAAs, e.g. of size 45 − 55 mm. Furthermore, all discussed approaches are unable to make any prediction about the future development of the AAA, such that the rupture risk assessment only holds for the point in time of data generation. In addition to that, the biomechanical model does not take into account factors like growth, calcifications and surrounding organs, which might be important for the analysis.

## 5 Conclusion

We presented a novel data-informed, highly personalized, probabilistic framework for the quantification of abdominal aortic aneurysm (AAA) rupture risk and demonstrated competitive performance in comparison to existing approaches. Our framework results in the calculation of a rupture risk index, ${\mathbb{P}}_{rupt}$, which can be introduced as a relevant additional piece of information in the clinical decision process for AAA cases that are not or not unambiguously covered by existing guidelines and recommendations. In view of our results it is suggested to incorporate personalized, or at least cohort-based, statistical information and choose a probabilistic approach for the biomechanical rupture risk assessment. Deterministic indices were shown to be less accurate and do not account for possible sensitivities due to uncertain vessel wall quantities.

In order to advance this framework to a clinical application, several further aspects need to be examined. Challenges lie especially in the fully automatic segmentation of the CT imaging data, which at the moment requires manual steps by a trained expert and can be time consuming. In view of the limitations discussed in Section 4, a larger, randomized study with risk factor matched groups is desirable to confirm this study’s findings regarding its clinical use. Future work will also address how further model parameters such as the blood pressure influence the rupture risk index and whether this quantity should be treated probabilistically as well. Lastly, to be able to make predictions over time, it is required to incorporate AAA growth [46, 47] into the framework and analyze its effect on the biomechanical rupture risk assessment.

## A Appendix

### A.1 Multi-output Gaussian process regression

The data generation process for log**Θ** is assumed to underly a function $\text{log}\tilde{\Theta }\left(\xi \right)$ that is contaminated by additive Gaussian noise, such that

$$\begin{array}{c} \log\Theta \left(\xi \right)=\log\tilde{\Theta }(\xi )+\epsilon . \end{array}$$ (31)

It is further postulated that the vector $\text{log}\tilde{\Theta }=[\text{log}\tilde{t},\text{log}\tilde{\alpha },\text{log}\tilde{\beta },\text{log}{\tilde{\sigma }}_{\gamma }{]}^{T}∼N\left(0,\Omega \right)$ follows a multivariate Gaussian distribution with the positive semi-definite covariance matrix $\Omega \in {\mathbb{R}}^{4\times 4}$ and it is assumed that ∈∼N(0,S), with the diagonal matrix **S** and noise levels $S_{dd}\in {\mathbb{R}}_{+}(d=1\ldots 4)$. Demanding that every entry of the vector $\text{log}\tilde{\Theta }(\xi )$ corresponds to the same zero mean Gaussian process with covariance function *k*(***ξ***, ***ξ***′), i.e.

$$\begin{array}{c} \log\tilde{t}\left(\xi \right),\log\tilde{\alpha }\left(\xi \right),\log\tilde{\beta }\left(\xi \right),\log{\tilde{\sigma }}_{\gamma }\left(\xi \right)∼GP(0,k(\xi ,\xi^{\prime })), \end{array}$$ (32)

$\text{log}\tilde{\Theta }(\xi )$ can be expressed as a multivariate Gaussian process [39]

$$\begin{array}{c} \log\tilde{\Theta }\left(\xi \right)∼MGP(0,k(\xi ,\xi^{\prime }),\Omega ). \end{array}$$ (33)

As a result, the collection $\{\text{log}{\tilde{\Theta }}_{i}{\}}_{i=1}^{n_{data}}$ follows a matrix-variate Gaussian distribution

$$\begin{array}{c} {\left[\log{\tilde{\Theta }}_{1},\ldots ,\log{\tilde{\Theta }}_{n_{\text{data}}}\right]}^{T}∼MN(0,K,\Omega ), \end{array}$$ (34)

with the covariance matrix *K* and entries *K*_*ij*_ = *k*(***ξ***_*i*_, ***ξ***_*j*_), modeling the covariance between two inputs ***ξ***_*i*_ and ***ξ***_*j*_. Expressing the matrix Gaussian distribution as a multivariate Gaussian distribution and incorporating the additive noise **ϵ**, one obtains

$$\begin{array}{c} \text{vec}\left(\log\hat{\Theta }\right)={\left[\log\Theta_{1}^{T},\ldots ,\log\Theta_{n_{\text{data}}}^{T}\right]}^{T}∼N(0,\Omega ⊗K+S⊗I_{n_{\text{data}}}), \end{array}$$ (35)

where ⊗ denotes the Kronecker product and $I_{n_{data}}$ the *n*_data_ × *n*_data_ identity matrix. For our purposes, we choose the covariance function

$$\begin{array}{c} k\left(\xi ,\xi^{\prime }\right)=\zeta_{1}+\zeta_{2}\xi^{T}\xi^{\prime }+\zeta_{3}\exp[-\zeta_{4}{\left(\xi -\xi^{\prime }\right)}^{T}(\xi -\xi^{\prime })], \end{array}$$ (36)

with hyperparameters *ζ*_1_, *ζ*_2_, *ζ*_3_ and *ζ*_4_. Following [37–39], the matrix *Ω* is parameterized via the entries *L*_*ij*_ of a Cholesky decomposition Ω = **LL**^T^. Together with the noise parameters from the matrix **S**, this results in the hyperparameter vector

$$\begin{array}{c} \zeta ={\left[\zeta_{1},\zeta_{2},\zeta_{3},\zeta_{4},S_{11},S_{22},S_{33},S_{44},L_{11},L_{22},L_{33},L_{44},L_{21},L_{31},L_{41},L_{32},L_{42},L_{43}\right]}^{T}, \end{array}$$ (37)

where $\zeta_{1},\zeta_{2},\zeta_{3},\zeta_{4},S_{11},S_{22},S_{33},S_{44},L_{11},L_{22},L_{33},L_{44}\in {\mathbb{R}}_{+}$ and $L_{21},L_{31},L_{41},L_{32},L_{42},L_{43}\in \mathbb{R}$. The predicted mean for an arbitrary point ***ξ***^⋆^ becomes

$$\begin{array}{c} \mu_{\log\Theta }(\xi^{⋆})={\left(\Omega ⊗k^{⋆}\right)}^{T}{\left(\Omega ⊗K+S⊗I_{n_{\text{data}}}\right)}^{-1}\text{vec}\left(\log\hat{\Theta }\right) \end{array}$$ (38)

and the predicted covariance

$$\begin{array}{c} \Sigma_{\log\Theta }(\xi^{⋆})=\Omega \;k\left(\xi^{⋆},\xi^{⋆}\right)+S-{\left(\Omega ⊗k^{⋆}\right)}^{T}{\left(\Omega ⊗K+S⊗I_{n_{\text{data}}}\right)}^{-1}(\Omega ⊗k^{⋆}), \end{array}$$ (39)

where **k**^⋆^ denotes the vector of covariance function evaluations between ***ξ***^⋆^ and the data $\{\xi_{i}{\}}_{i=1}^{n_{data}}$, i.e.

$$\begin{array}{c} k^{⋆}={\left[k\left(\xi^{⋆},\xi_{1}\right),\ldots ,k\left(\xi^{⋆},\xi_{n_{\text{data}}}\right)\right]}^{T}. \end{array}$$ (40)

Finally, the log marginal likelihood is

$$\begin{array}{c} \begin{array}{cc} L(\zeta )=\log p\left(\log\hat{\Theta }\;|\;{\left\{\xi_{i}\right\}}_{i=1}^{n_{\text{data}}}\right)= & -\frac{1}{2}\log\left|\Omega ⊗K+S⊗I_{n_{\text{data}}}\right|\\ & -\frac{1}{2}\text{vec}{\left(\log\hat{\Theta }\right)}^{T}{\left(\Omega ⊗K+S⊗I_{n_{\text{data}}}\right)}^{-1}\text{vec}\left(\log\hat{\Theta }\right)\\ & -2n_{\text{data}}\log2\pi \end{array} \end{array}$$ (41)

and can be optimized with respect to its hyperparameters ***ζ***.

### A.2 Kriging surrogate incorporating explicit basis functions

Kriging can be regarded as a special case of a Gaussian process, where data points are assumed noise-free to interpolate the high-fidelity model at the provided high-fidelity evaluations. To find an adequate function for this purpose, we use a Kriging interpolation model that incorporates explicit basis functions as described in [42]. Using such a model, it is possible to exactly represent functions that can be described by the provided basis. Ensuring positive predictions via a log transformation, we approximate the high-fidelity model as

$$\begin{array}{c} \log\sigma_{\text{vm}}^{\text{max}}\left(\theta \right)\approx \log{\tilde{\sigma }}_{\text{vm}}^{\text{max}}\left(\theta \right)+h{\left(\theta \right)}^{T}\eta , \end{array}$$ (42)

where $\text{log}{\tilde{\sigma }}_{vm}^{max}(\theta )∼GP\left(0,k(\theta ,\theta^{\prime })\right)$ is a zero mean Gaussian process with covariance function ***k***(***θ***, ***θ***′) and ***h***(***θ***) denotes the chosen basis functions with coefficients ***η***. A simple squared exponential kernel

$$\begin{array}{c} k\left(\theta ,\theta^{\prime }\right)=\zeta_{1}\exp[-\frac{1}{2}{\left(\theta -\theta^{\prime }\right)}^{T}\Lambda^{-1}(\theta -\theta^{\prime })] \end{array}$$ (43)

is chosen, where the matrix $\Lambda =\text{diag}\left(\zeta_{2},\zeta_{3},\zeta_{4}\right)\in R^{3\times 3}$ is diagonal, leading to the vector of hyperparameters *ζ* = [*ζ*_1_, *ζ*_2_, *ζ*_3_, *ζ*_4_]^T^, with $\zeta \in R_{+}^{4}$. Furthermore, trilinear basis functions, i.e.

$$\begin{array}{c} h\left(\theta \right)={\left[1,t,\alpha ,\beta ,t\alpha ,t\beta ,\alpha \beta ,t\alpha \beta \right]}^{T} \end{array}$$ (44)

are employed. Assuming a Gaussian prior for the coefficients, η∼N(b,B), this results in the Gaussian process

$$\begin{array}{c} \log{\tilde{\sigma }}_{\text{vm}}^{\text{max}}\left(\theta \right)+h{\left(\theta \right)}^{T}\eta ∼GP(h{\left(\theta \right)}^{T}b,k\left(\theta ,\theta^{\prime }\right)+h{\left(\theta \right)}^{T}Bh\left(\theta^{\prime }\right)). \end{array}$$ (45)

The dependence on the prior parameters ***b*** and *B* can be resolved, if a vague prior for ***η*** is chosen, i.e. if the limiting case is considered, where *B*^−1^ approaches the zero matrix 0. In that case, the predicted mean for a new point ***θ***^⋆^ becomes

$$\begin{array}{c} \begin{array}{cc} \mu_{\log\sigma_{\text{vm}}^{\text{max}}}\left(\theta^{⋆}\right) & =k^{{⋆}^{T}}K^{-1}\hat{\sigma }+r^{T}\bar{\eta }=\\ & =\mu_{\log{\tilde{\sigma }}_{\text{vm}}^{\text{max}}}\left(\theta^{⋆}\right)+r^{T}\bar{\eta } \end{array} \end{array}$$ (46)

and the predicted variance

$$\begin{array}{c} \begin{array}{cc} \delta_{\log\sigma_{\text{vm}}^{\text{max}}}^{2}\left(\theta^{⋆}\right) & =k\left(\theta^{⋆},\theta^{⋆}\right)-k^{{⋆}^{T}}K^{-1}k^{⋆}+r^{T}{\left(HK^{-1}H^{T}\right)}^{-1}r=\\ & =\delta_{\log{\tilde{\sigma }}_{\text{vm}}^{\text{max}}}^{2}\left(\theta^{⋆}\right)+r^{T}{\left(HK^{-1}H^{T}\right)}^{-1}r, \end{array} \end{array}$$ (47)

where $\bar{\eta }={\left(HK^{-1}H^{T}\right)}^{-1}HK^{-1}\hat{\sigma }$, ***r*** = ***h***^⋆^ − *HK*^−1^ ***k***^⋆^ and $\hat{\sigma }={\left[\text{log}\sigma_{vm}^{max}(\theta_{1}),\ldots ,\text{log}\sigma_{vm}^{max}(\theta_{n_{eval}})\right]}^{T}$ is the vector of *n*_eval_ model evaluations. *K* is the data covariance matrix between all $\hat{\theta }={\left[\theta_{1},\ldots ,\theta_{n_{eval}}\right]}^{T}$ training data points such that

$$\begin{array}{c} K_{ij}=k\left(\theta_{i},\theta_{j}\right). \end{array}$$ (48)

Moreover, ***k***^⋆^ is a vector with the covariances between training and test points, i.e.

$$\begin{array}{c} k^{⋆}={\left[k\left(\theta^{⋆},\theta_{1}\right),\ldots ,k\left(\theta^{⋆},\theta_{n_{\text{eval}}}\right)\right]}^{T}, \end{array}$$ (49)

*H* a matrix containing vectors **h**(*θ*) at all training data points and ***h***^⋆^ = ***h***(*θ*^⋆^). It is interesting to note, how the terms in Eqs (46) and (47) consist of a contribution from the zero mean Gaussian process predicted mean and variance, $\mu_{\text{log}{\tilde{\sigma }}_{vm}^{max}}(\theta^{⋆})$ and $\delta_{\text{log}{\tilde{\sigma }}_{vm}^{max}}^{2}(\theta^{⋆})$, and additional terms involving the provided basis functions, respectively. Finally, the marginal log likelihood is

$$\begin{array}{c} L(\zeta )=\log p\left(\hat{\sigma }|\hat{\theta }\right)=-\frac{1}{2}{\hat{\sigma }}^{T}K^{-1}\hat{\sigma }+\frac{1}{2}{\hat{\sigma }}^{T}C\hat{\sigma }-\frac{1}{2}\log|K|-\frac{1}{2}\log\left|A\right|-\frac{n_{\text{eval}}-m}{2}\log2\pi , \end{array}$$ (50)

where *A* = *HK*^−1^ *H*^T^ *C* = *K*^−1^ *H*^T^ *A*^−1^ *HK*^−1^ and *m* is the rank of *H*^T^. Maximizing the marginal log likelihood, optimal values for the hyperparameters ***ζ*** of the Kriging covariance model (cf. Eq (43)) and for the provided $\hat{\sigma }$ and $\hat{\theta }$ can be determined.

## Supporting information

S1 Table Correlation analysis. Computed correlations between all non-invasive and invasive AAA vessel wall properties using Spearman’s rank correlation coefficient.
(CSV)

S2 Table Non-invasive data. Dataset containing the eight non-invasive AAA vessel wall properties: maximum AAA diameter, maximum thrombus thickness, AAA length, subrenal diameter, thrombocytes, hemoglobin, mean corpuscular hemoglobin (MCH), mean corpuscular volume (MCV). All values are provided in their respective unit as specified in Table 2. The first column is a consecutive number for the individual patients and allows for the identification of data that was collected from the same patient.
(CSV)

S3 Table Invasive data. Dataset containing the four invasive AAA vessel wall properties: wall thickness, alpha stiffness, beta stiffness, wall strength. All values are provided in their respective unit as specified in Table 1. The first column is a consecutive number for the individual patients and allows for the identification of data that was collected from the same patient.
(CSV)

S4 TableROC values.Computed true positive rates (TPR), false positive rates (FPR) and corresponding threshold values for ${\mathbb{P}}_{rupt}$.(CSV)
